# Central auditory processing is altered after traumatic brain injury in Tanzanian adults

**DOI:** 10.3389/fnins.2025.1720978

**Published:** 2025-12-12

**Authors:** Jonathan D. Lichtenstein, Christopher E. Niemczak, Abigail Fellows, Lindsay J. Wood, Shufaa Masoud, Alexis Rooney, Monika Adhikari, Albert Magohe, Jay C. Buckey

**Affiliations:** 1 Department of Psychiatry, Geisel School of Medicine at Darmouth, Hanover, NH, United States; 2 Department of Pediatrics, Geisel School of Medicine at Dartmouth, Hanover, NH, United States; 3 The Dartmouth Institute, Geisel School of Medicine at Dartmouth, Hanover, NH, United States; 4 Department of Medicine, Dartmouth-Hitchcock Medical Center, Lebanon, NH, United States; 5 Space Medicine Innovations Lab, Geisel School of Medicine, Hanover, NH, United States; 6 Dartmouth College, Hanover, NH, United States; 7 Muhimbili University of Health and Allied Sciences, Dar es Salaam, Tanzania; 8 Department of Audiology, Montclair State University, Montclair, NJ, United States

**Keywords:** assessment, central auditory processing, LMICs (low and middle income countries), sub-Sahara Africa (SSA), traumatic brain injury

## Abstract

Traumatic brain injury (TBI) damages pathways throughout the brain and is a significant global health concern, particularly in low- and middle-income countries, where the incidence is high and long-term deficits are prevalent. This study explores the utility of central auditory processing (CAP) testing as a marker of previous TBI. Seventy individuals with a history of moderate to severe TBI (msTBI) were matched by age and sex to 46 healthy controls in Dar es Salaam, Tanzania. Participants underwent comprehensive behavioral CAP testing, including tests of speech-in-noise ability, temporal resolution, and dichotic listening. Multivariate logistic regression showed the Triple Digit Test (TDT) (*p* < 0.001) significantly predicted msTBI status, independent of age and peripheral hearing ability. Elastic net modeling supported these findings, highlighting TDT performance as the most robust predictor of msTBI history. A history of msTBI is associated with poorer CAP performance, particularly on speech-in-noise tests. These tests could serve as accessible, resource-efficient tools for assessing brain function related to TBI in clinical and resource-limited settings. Studies in larger, more diverse populations are needed to explore their predictive utility for long-term cognitive outcomes after TBI.

## Introduction

Traumatic brain injury (TBI) presents a significant and increasing global health challenge due to high incidence, prevalence, and disparities in care following injury (Pavlovic et al., 2019). Current global TBI incidence is estimated at 27 to 69 million per year (Williamson and Rajajee, 2023), with low- and middle-income countries (LMICs) experiencing higher incidence, morbidity, and mortality (GBD 2016 Traumatic Brain Injury and Spinal Cord Injury Collaborators, 2019). Sub-Saharan African countries are particularly affected, with a significantly higher burden compared to the global average as well as an upward trending incidence in recent years (Adegboyega et al., 2021). While advances in TBI treatment have lowered TBI-related death rates, these improvements have given rise to a growing population of individuals living with TBI-related deficits. These individuals are at high risk for numerous health, social, and economic challenges (National Academies of Sciences, Engineering, and Medicine, Health and Medicine Division, Board on Health Care Services, Board on Health Sciences Policy, Committee on Accelerating Progress in Traumatic Brain Injury Research and Care, 2022; Khellaf et al., 2019; Galgano et al., 2017) including long-term cognitive and sensory impairments.

One promising approach to detecting and monitoring TBI-related deficits is through assessing auditory function, as the auditory system—particularly central auditory processing—can be sensitive to brain injuries (Nölle et al., 2004; Papesh et al., 2018; Buriti and Gil, 2022). The auditory system’s distributed neural networks throughout the brainstem and cortex make it particularly vulnerable to the diffuse axonal injury characteristic of TBI (Fino et al., 2022; Sergio et al., 2020; Alnawmasi et al., 2019). Speech perception in noise requires rapid integration of acoustic information and relies on attention, working memory, and executive functions—cognitive domains commonly affected by TBI (Amanipour et al., 2016; Ouyang et al., 2017; Thompson et al., 2018; Gallun et al., 2012; Kraus et al., 2016; Vander Werff and Rieger, 2017; Rauterkus et al., 2021; Kraus et al., 2017; Turgeon et al., 2011; Bergemalm and Lyxell, 2005; Cockrell and Gregory, 1992; Vander Werff and Rieger, 2019a, b; Kozin et al., 2021; Vander Werff, 2016). Central auditory test results, such as performance on speech perception in noise tests, particularly in individuals with normal peripheral hearing, could serve as potential markers for TBI, aiding in early diagnosis and long-term monitoring. Detecting lasting TBI-related deficits is imperative for improving outcomes across domains, although significant barriers to receiving such care exist, particularly in LMICs (Buh et al., 2023).

TBI encompasses any sign of brain damage or disruption in brain function resulting from an external force, with moderate to severe TBIs (msTBIs) causing significant neurological disabilities (Pavlovic et al., 2019). TBI is a complex phenomenon comprised of multiple underlying mechanisms of injury such as contact/inertial forces and cellular response to injury (Khellaf et al., 2019; Galgano et al., 2017; White-Schwoch et al., 2020), which can lead to diffuse neurological damage. The immediate physical impact results in brain tissue disruption such as axonal shearing, hemorrhage, contusion, and blood vessel damage, often followed by secondary injury, commonly resulting in secondary cellular responses that can lead to brain cell death, tissue damage, and atrophy (Pavlovic et al., 2019; Galgano et al., 2017). These injuries can lead to structural and functional alterations to the brain and disrupt cognitive function. Axonal shearing, or in severe cases, diffuse axonal injury, causes alterations in white matter with subsequent pervasive and diffuse disruptions to various cognitive functions, including slowed processing speed and poor working memory, as well as global cognitive impairment. Further cognitive impacts include deficits in attention, concentration, and mental flexibility (Halalmeh et al., 2024). Clinical manifestations of TBI vary widely across physical, cognitive, and affective domains (Pavlovic et al., 2019). Long-term outcomes associated with msTBI include persistent challenges with activities of daily living, need for personalized care across the lifespan, and increased risk for neurocognitive decline and neurodegenerative disease with age (McCrea et al., 2021).

Detection and care for msTBI requires ongoing and sometimes costly services that are not always available in LMICs (Pavlovic et al., 2019; Halalmeh et al., 2024). Importantly, while complete cognitive recovery following msTBI is rare, many individuals experience some improvement in cognitive functioning, particularly within approximately the first year following injury. Several longitudinal outcome studies suggest that as many as 20% of msTBI survivors will recover functional independence within 5 years post injury (McCrea et al., 2021). Research supports neurorehabilitation as an essential part of improving TBI outcomes (Buh et al., 2023). Thus, neuropsychological assessment represents an integral component of care following TBI given the importance of assessment and monitoring of cognitive functioning to inform intervention planning throughout recovery (Buh et al., 2023; Halalmeh et al., 2024; Torregrossa et al., 2023).

While a comprehensive approach to neuropsychological assessment with TBI patients is the gold standard for evaluating cognitive sequelae after TBI, significant barriers limit access to such care globally, particularly in resource limited settings such as LMICs (Buh et al., 2023; Torregrossa et al., 2023). Comprehensive neuropsychological assessment is often costly and time-intensive, requiring highly specialized training for administration and interpretation. In addition, most neuropsychological assessments are affected by culture, and significant challenges persist in finding linguistically and culturally appropriate normative samples (Robertson et al., 2009). Further, normative data may not generalize across populations.

In contrast, central auditory processing (CAP) focused tests are time-efficient compared to traditional neurocognitive batteries, easy to administer, and far less reliant on culture and education (Niemczak et al., 2021). Although CAP tests cannot replace comprehensive neuropsychology assessment, they may serve as adjunct tools, especially in LMICs. While peripheral auditory function (i.e., hearing sensitivity, such as raising your hand to soft tone) is one component of auditory processing, CAP tests (i.e., complex auditory processing, such perceiving speech in background noise) require rapid and diffuse cortical processing (Joseph et al., 2024), which may be affected by the diffuse nature of TBI itself. Processing auditory information offers a unique window into brain function because it is neurologically demanding (Niemczak et al., 2021; Joseph et al., 2024). After sound waves are converted into nerve signals by the cochlea, the brain must quickly process an array of complex information while filtering out background noise, extracting timing information, distinguishing relevant frequencies, and ascertaining the meaning of the content (Niemczak et al., 2021). These processes rely on neural pathways located throughout the brainstem and into the cortex that integrate with high level linguistic and cognitive systems that require adequate processing speed, working memory, and attention (Niemczak et al., 2021).

Advances in technology have made it possible to test central auditory processing in austere settings without sound attenuating booths. Specifically, the WAHTS headset has sound attenuation comparable to a sound-booth (Kulinski et al., 2022). The system is portable, wireless, and easy to train examiners on how to use. Further, literature supports a correlation between central auditory function, as measured by tests of speech perception in background noise, and neurocognitive performance (Niemczak et al., 2021). Previous work exploring the use of CAP tests in cognitive assessment of individuals living with HIV evidenced a strong correlation between speech-in-noise performance and cognitive measures including learning and working memory measures (Niemczak et al., 2021). A follow up longitudinal study demonstrated the capacity for CAP tests to track cognitive function over time in individuals living with and without HIV (Niemczak et al., 2021). Importantly, results are generally independent of peripheral hearing functioning, suggesting a true relationship between poor performance on CAP tests and cognitive impairment related to central nervous system damage (White-Schwoch et al., 2020). This suggests that CAP tests may track cognitive functioning in disorders and injuries affecting the central nervous system, such as TBI (Niemczak et al., 2021). Pairing these associations between CAP and cognition with the real-world application of CAP testing via the WAHTS system, individuals in resource-limited settings may benefit from increased access to assessment of brain-based functioning.

While recent evidence shows congruent findings in the mild TBI population, with data indicating auditory deficits in adults following cerebral concussion, little data are available regarding the use of CAP tests in adults with moderate to severe TBIs (Hoover et al., 2017; de Godoy et al., 2022). The current study proposes CAP testing as an auxiliary or alternative method for measuring neural processing problems in individuals with msTBI, particularly in limited resource settings. To demonstrate this link, we measured CAP test performance in a group of people with msTBI compared to individuals without TBI. We hypothesized that central auditory test performance would be significantly worse in individuals with a history of msTBI compared to controls. We also hypothesized that CAP test performance would be able to predict those with a history of msTBI.

## Materials and methods

Participants in this study were specifically recruited due to a history of self-reported msTBI. The matched control group was derived from an ongoing longitudinal study examining the neurocognitive effects of HIV on the central auditory system in adults living in Dar es Salaam, Tanzania. To form a matched control group, subjects from the group in the longitudinal study living without HIV were matched by age and sex to the TBI participants. Seventy individuals with a history of msTBI (mean age: 32.4 years, 62% male) were enrolled and were matched with 46 control subjects (mean age: 31.6 years, 59% male).

The research protocol for this study was approved by the institutional review boards of Dartmouth College and the Muhimbili University of Health and Allied Sciences. Written informed consent was obtained from all participants before their inclusion. Testing took place at the Infectious Disease Center in Dar es Salaam, Tanzania. To ensure the accuracy of the analysis and control for factors that could influence central auditory and cognitive function, participants with abnormal hearing sensitivity (>25 dB HL from 0.5 to 8 kHz) or abnormal middle ear function were excluded. Detailed demographics of the study population can be found in Table 1.

**Table 1:** Demographics.

Variable	Control (*n* = 46)	msTBI (*n* = 70)	*p*-value
Age in years (SD)	31.6 (10.9)	32.4 (10.0)	0.696
Percent male (%)	59%	62%	0.768
Pure tone average (SD)	4.13 (7.67)	1.28 (6.58)	0.080
Total problems reported due to msTBI (SD)	0.00 (0)	1.22 (0.90)	<0.001
Months since msTBI (SD)	–	78.3 (97.2)	–
Hospital admission days (SD)	–	16.5 (32.6)	–
Loss of consciousness hours (SD)	–	26.6 (40.6)	–

msTBI, moderate-to-severe traumatic brain injury.

Auditory testing was conducted using Creare’s Wireless Auditory Hearing Testing System (WAHTS), which features speakers mounted in highly noise-attenuating ear cups. The left and right ear signals are processed to simulate different locations of the speech and noise sources. This system outperforms all currently available commercial hearing test devices, as demonstrated by an independent laboratory evaluation following relevant ANSI standards (Meinke et al., 2017). Prior to the audiological assessment, patients underwent an otoscopic examination, with cerumen removal as needed. Tympanometry at 226 Hz was performed on both ears using a Madsen Otoflex 100 (GN Otometrics, Denmark) to confirm normal middle ear function. Individuals with abnormal tympanograms (Type B or C) were referred for treatment and re-evaluation. Peripheral hearing ability was assessed at 0.5, 1.0, 2.0, 4.0, 6.0, and 8.0 kHz using a Békésy-like tracking procedure, as previously described (Maro et al., 2014). Peripheral hearing thresholds above 8.0 kHz were not tested. Normal hearing was defined as thresholds of 25 dB HL or better for each ear across these frequencies, and pure tone averages (PTA) were calculated by averaging the thresholds across all measured frequencies. PTA for this study was derived 500, 1,000, 2,000, and 4,000 Hz (Table 2).

**Table 2:** Mechanisms and current reported problems.

Injury mechanisms (% of msTBI sample)	Reported problems (% of sample)
Motor vehicle collision (51)	Headaches (51)
Undefined (23)	Dizziness (16)
Fall (10)	Noise intolerance (14)
Assault (7)	Other (14)
Road traffic accident (5.2)	Short-term memory loss (7)
Bicycle accident (1.4)	Balance issues (6)
Animal encounter (1.4)	Lower limb numbness (6)
–	Vision issues (3)
–	Sluggish speech (3)
–	Seizures (1.4)
–	Nystagmus (1.4)

msTBI, moderate-to-severe traumatic brain injury.

Four central auditory tests were administered to each participant: the Triple Digit Test (TDT), the Hearing in Noise Test (HINT), the Gap Detection Test (gap), and the Staggered Spondaic Words test (SSW). All CAP tests were conducted using the WAHTS in Kiswahili, the native language of all participating subjects. The entire central auditory battery took approximately 40 min.

The TDT (Triple Digit Test) and HINT (Hearing in Noise Test) were both designed to evaluate speech-in-noise perception. The methods used in this study have been previously detailed by Niemczak, Lichtenstein, et al. (2021) and are summarized here. In the TDT, participants were presented with 30 sets of three-digit triplets (e.g., 2-8-1, translated as mbili-nane-moja) binaurally. Recognition of these digit triplets was assessed in the presence of Schroeder-phase masking noise using an adaptive testing paradigm (Niemczak et al., 2021). The test began with an initial signal-to-noise ratio (SNR) of 0 dB, with the masker set at a fixed level of 75 dB sound pressure level (SPL). The SNR was adjusted adaptively after each presentation, with 0.5 dB decreases for each correct digit response and 2 dB increase for every incorrect digit response. The average SNR of the last six trials was used as the speech reception threshold. The HINT, in contrast, uses sentence stimuli instead of digit triplets. The HINT tests speech recognition of sentences in background noise with varying spatial locations (simulated virtually) from 0 degrees (Noise-*Front*) or −/+ 90 degrees azimuth (*Left, Right*). For each condition, a different, randomized list of 20-sentences was presented. Testing was conducted using an adaptive paradigm across three conditions: Noise Front, Noise Right, and Noise Left. In each condition, a different set of sentences was presented with speech-shaped masking noise at a constant level of 75 dB SPL. The level of each sentence was adjusted adaptively based on whether the participant correctly repeated the previous sentence. The primary outcome variable for the HINT was the average SNR for each noise condition using the following formula to average across spatial locations (*2 x Front + (Left + Right)*)/4. The goal was to determine the lowest SNR at which sentences could be repeated reliably.

The gap detection test assesses auditory temporal resolution, as previously described (Niemczak et al., 2021; Maro et al., 2014). During this test, participants listened to a block of white noise, set at 65 dB, that contained a brief gap (a momentary pause in the noise) and pressed a button as soon as they detected the gap (Buss et al., 2017). An adaptive algorithm was used to determine each participant’s gap detection threshold, defined as the shortest gap they could reliably detect. The test began with a 20 ms gap and continued until the participant’s gap threshold was determined, based on the average of their five shortest reliably detected gaps. The relationship between gap detection accuracy and gap length was then plotted, showing the percentage of correctly detected gaps at varying gap durations. This curve was fitted using the Hill equation, which enabled the calculation of the gap duration at which participants detected 50% of the gaps correctly (Hill, 1910; Niemczak et al., 2023). These threshold values were subsequently used for analysis.

The SSW (Staggered Spondaic Word) test evaluates dichotic auditory processing. In this test, two spondaic words (e.g., *vuna-ngano* and *pata-pesa*, roughly translating to “harvest-wheat” and “earn-money”) are presented such that the first syllable of one word and the second syllable of the other are delivered simultaneously to both ears with speech-shaped masking noise at a constant level of 65 dB SPL (Katz and Smith, 1991). Participants are instructed to repeat all four words in the order they heard them. Each syllable is scored as correct or incorrect based on the participant’s response.

### Statistical analysis

Logistic regression and elastic net regression were used to assess the relationship between msTBI and auditory measures. Data were analyzed and plotted using MATLAB® 2020b. To standardize variables for analysis, z-scores were computed for all central auditory tests and pure tone average (PTA) using the entire cohort. The mean value of each z-score variable was calculated, and missing values were imputed with these means (z-score = 0.0) to maintain data integrity. The number of missing values imputed were as follows: 3 for TDT, 2 for SSW, 4 for gap, 3 for HINT, and 1 for PTA. For the HINT, 8 subjects had missing data on one of the left or right localizations conditions. For these subjects, if the PTA was within 10 dB HL between ears, the localization score was duplicated for the missing value. These standardized scores allowed for a uniform comparison between measures, facilitating cross-group analysis. A composite score, the Global Central auditory Score (GCS), was calculated as the mean of the z-scores for all central auditory tests. This GCS score served as a comprehensive metric of participant cognitive and auditory performance.

A multivariate logistic regression was used to predict participant group (msTBI vs. no-TBI) using the following model: Group ~ TBI + HINT + SSW + Gap + Age + PTA. We used bootstrapping to create a performance metric for the regression (area under the curve; AUC). An additional multivariate logistic regression model was implemented using just GCS as a predictor along with age and PTA. Because the results of the various central auditory tests could be correlated, we also used elastic-net logistic regression (*α* = 0.75) to model the group inclusion (msTBI = 1, no history = 0) from z-scored central auditory predictors as well as age and PTA. Elastic net regression is particularly suited for handling multicollinearity among predictors, such as CAP tests, and selecting relevant features that predict TBI group. For each fit, the penalty *λ* was chosen by 10-fold cross-validation (minimum cross-validated mean squared error (MSE)). To evaluate variable stability, we performed 100 bootstrap resamples (sampling participants with replacement). In each bootstrap, we refit the elastic-net and recorded whether each predictor’s coefficient was non-zero and whether it was positive or negative. Selection frequency was defined as the proportion of bootstraps in which a predictor was selected. As a negative control, we included an additional Gaussian “Noise” predictor to benchmark spurious selection. We reported selection frequencies at λmin.

## Results

The multivariate logistic regression (Table 3) showed that the TDT emerged was a significant predictor with an odds ratio of 2.78 (*p* < 0.001, Cohen’s *d* = 0.52), indicating that poorer scores (i.e., higher SNR) on this test substantially increased the likelihood of being in the msTBI group. The HINT also contributed as a significant predictor, with an odds ratio of 1.72 (*p* = 0.045, Cohen’s *d* = 0.31). The bootstrapped AUC estimate was 0.78 with a confidence interval of 0.71 to 0.86. In a separate multivariate logistic regression (Table 4), the GCS was also a significant predictor, with an odds ratio of 4.30 (*p* < 0.001, Cohen’s *d* = 0.50), highlighting its potential as an effective marker for distinguishing between groups. In contrast, variables such as SSW, gap, PTA and age did not significantly predict msTBI group status.

**Table 3:** Multivariate logistic regression: prediction of msTBI group with individual CAP variables.

Outcome	Predictor	Odds ratio (CI)	SE	tStat	*p* value	Cohen’s *d*
TBI group	PTA	0.985 (0.618, 1.571)	0.238	−0.061	0.951	0.009
	Age	0.868 (0.950, 1.043)	0.023	−0.165	0.868	0.025
	TDT	**2.776 (1.535, 5.021)**	**0.302**	**3.378**	**<0.001**	**0.515**
	HINT	**1.722 (1.012, 2.931)**	**0.271**	**0.004**	**0.045**	**0.306**
	SSW	1.797 (0.518, 6.228)	0.634	0.9241	0.355	0.141
	Gap	0.873 (0.518, 1.472)	0.2664	−0.506	0.612	0.077

CI, confidence interval; PTA, pure tone average; TDT, triple digit test; HINT, hearing in noise test; SSW, staggered spondaic words; Gap, gap detection test. Bold text corresponds to statistically significant variables.

**Table 4:** Multivariate logistic regression: prediction of msTBI group with GCS.

Outcome	Predictor	Odds ratio (CI)	SE	tStat	*p* value	Cohen’s *d*
msTBI group	PTA	0.961 (0.632, 1.461)	0.213	−0.185	0.852	0.028
	Age	0.990 (0.951, 1.031)	0.020	−0.461	0.644	0.070
	GCS	**4.390 (1.823, 10.56)**	**0.448**	**3.300**	**<0.001**	**0.503**

msTBI, moderate-to-severe traumatic brain injury; GCS, global CAP score; CI, confidence interval; PTA, pure tone average. Bold text corresponds to statistically significant variables.

**Figure 1:**
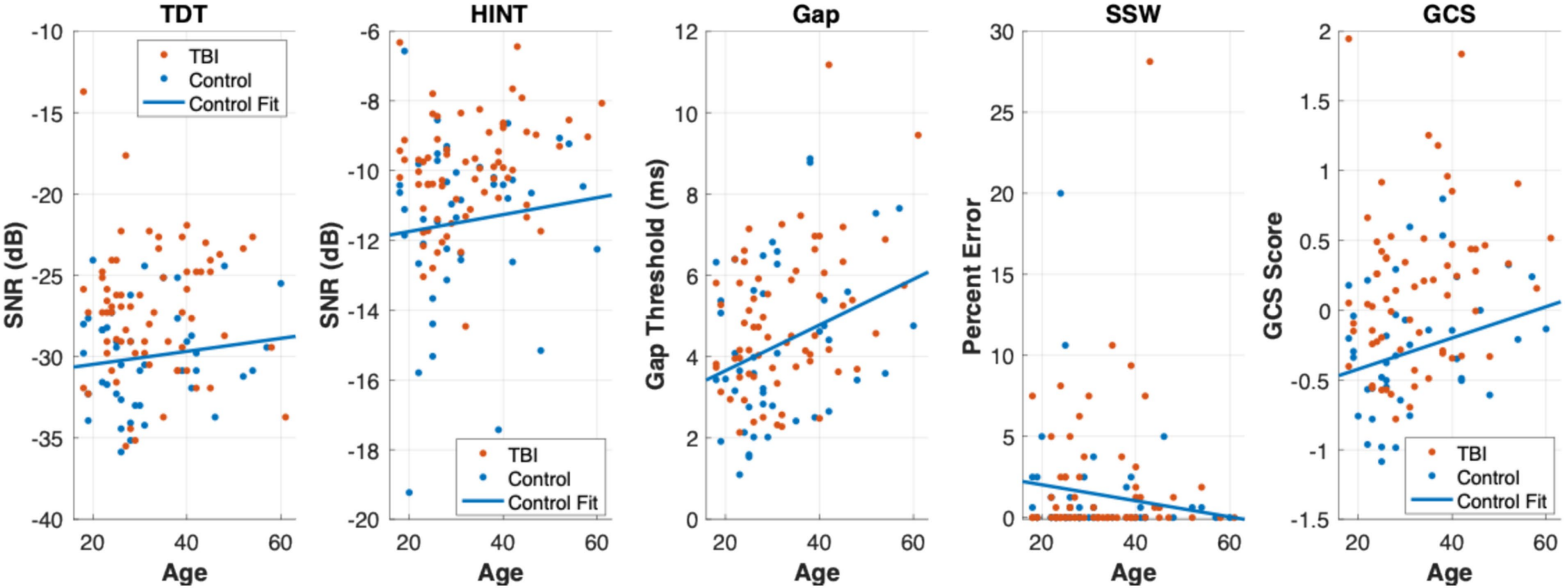
Scatter plots of central auditory processing tests across age. Scatter plots of the TDT, HINT, Gap, SSW, and GCS across age are displayed from left to right. TBI group participants are plotted in orange and controls are plotted in blue. A least-squares line was fit to the Controls to visualize the trajectory of these central auditory processing tests across age. In the TDT, HINT, and GCS plots, the majority of TBI group participants (orange points) can be viewed above the Controls fit line suggesting poorer scores across age on these tests.

Figure 1 shows scatter plots of all central auditory tests across age for both TBI (orange) and control (blue) groups. A least-squares line was fit to the control data to visualize the trajectory of auditory tests across age, but also to visually assess where the TBI subjects scored relative to the fit line. That is, TBI subjects were clearly visualized above the fit line (i.e., poorer scores across age) for the TDT, HINT, and GCS.

The elastic net model (Table 4) is shown below. The outcome was TBI group, and the predictors were age, TDT, HINT, SSW, gap, GCS, age and PTA. Results further supported the logistic regression findings, identifying the TDT as a consistent predictor. In 100 bootstrap resamples, elastic-net (*α* = 0.75) selected TDT and HINT in 99 and 91% of models. All coefficients for the TDT and HINT were positive indicating higher (worse) scores were associated with TBI. The finding reinforces speech-in-noise performance as a predictive factor for TBI classification. Importantly, age was not apredictor. These results underscore the significant role of specific central auditory tests, particularly the TDT and HINT in identifying individuals within the TBI group. These tests may serve as reliable markers for auditory and cognitive deficits associated with TBI (Table 5).

**Table 5:** Bootstrap elastic net model predictors.

Outcome	Predictor	Model predictor percentage	% Time predictor positive coefficient
TBI group	TDT	99%	99%
	Age	53%	22%
	HINT	91%	91%
	Random Noise	51%	6%
	Gap	45%	15%
	SSW	31%	31%
	GCS	12%	12%

TBI, traumatic brain injury; TDT, triple digit test; HINT, hearing in noise test; Gap, gap detection test; SSW, staggered spondaic words; GCS, global CAP score.

## Discussion

The msTBI and non-TBI groups differed significantly on central auditory test performance as measured by speech-in-noise tests. The msTBI group performed significantly worse on the TDT and HINT tests (i.e., speech-in-noise) compared to their healthy counterparts despite normal and matched peripheral hearing ability. The gap (i.e., temporal auditory processing) and SSW (i.e., dichotic auditory processing) results were not significantly different between groups. Thus, findings support a relationship between poor speech-in-noise performance and history of msTBI, suggesting the sensitivity of the TDT to residual deficits in individuals with msTBI, both post-acute and chronic.

Speech-in-noise difficulty is a common subjective complaint among patients with TBI [40, 48]. In one study, participants with a history of mild TBI (mTBI) were compared to a group with matched age and peripheral hearing ability. Results found that 84% of the TBI group reported difficulty understanding speech-in-noise compared to the matched group (9.1%), though there were no observed group differences on central auditory measures (Hoover et al., 2017). Other studies, however, have shown speech-in-noise problems in patients with mTBI, showing mild to moderate deficits on tasks with various target stimuli (i.e., words, sentences, and digits) (Hoover et al., 2017; Lander et al., 2024). For example, a very recent study found that adults with a history of mTBI performed significantly worse than controls on the low-cue Listening in Spatialized Noise-Sentence task (Hoover et al., 2017). A retrospective study found 60% of TBI patients showed deficits in speech-in-noise function on a variety of tests (Listening in Spatialized Noise-Sentence, Quick Speech in Noise, and the Bamford-Kowal-Bench Speech in Noise Test), with no significant link between severity of TBI (White et al., 2022). The current study contributes to the expanding body of evidence indicating speech-in-noise deficits in individuals with TBI, highlighting the need for further research to validate and translate these findings into clinical practice.

Studies with participants who experienced moderate-to-severe TBI have shown trends regarding significant difficulties in dichotic listening tasks (Levin et al., 1989; Meyers et al., 2002). A study by Levin et al. (1989) compared dichotic listening skills between a group of controls and participants who were hospitalized with a closed head injury. Consonant-vowel nonsense syllable pairs were used as target stimuli and results revealed a significant right ear advantage performance for msTBI (Levin et al., 1989). The Dichotic Word Listening Test has demonstrated sensitivity to cerebral dysfunction following TBI, with significantly lower scores observed in both children and adults with a history of TBI (Roberts et al., 1994). The dichotic auditory test used in the current study (SSW) did not reveal significant differences between groups. While our findings diverge from previous studies on dichotic auditory processing, the GCS score demonstrated significant differences between TBI groups using logistic regression but not elastic net regression. More work is needed to determine the value of using a composite CAP score for TBI populations.

Overall, previous studies showing speech-in-noise deficits in individuals with a history of TBI support the potential utility of such measures in capturing deficits secondary to frontal-subcortical dysfunction, similar to that commonly seen in TBI. Future research on auditory function and TBI should continue to focus on speech-in-noise ability but further examine the utility of a broader battery of auditory tests for providing a more comprehensive understanding of the relationship between frontal-subcortical dysfunction and central auditory processing.

In the present study, all individuals with msTBI recruited were outside the acute phase of injury (~one month), with a variable but lengthy average time elapsed since injury (~78 months since injury). This suggests CAP tests may be sensitive to dysfunction downstream from TBI, even when the injury is quite remote, tapping into the often “unseen” long-term sequalae associated with TBI. These findings may assist with identifying individuals with persistent neurological symptoms who may benefit from more intensive rehabilitation. The logistic regression results showed that CAP test performance predicts msTBI group membership even when accounting for both age and peripheral auditory function. This adds clinical meaning to our findings of central auditory dysfunction in individuals with msTBI, as outcome prediction is not due to expected age-related declines in cognition or peripheral hearing (i.e., presbycusis). This is particularly important in the context of prior work involving CAP testing and cognition, which has shown age to be a mediating factor in this relationship (Niemczak et al., 2021; Niemczak et al., 2021; Lichtenstein et al., 2022). If CAP tests can identify individuals with persistent neurological consequences from msTBI this could allow for targeted recommendations for the frequency and intensity of follow-up care and monitoring for individuals with msTBI. CAP tests may complement other assessment tools, as central auditory performance is rarely tested in TBI assessment protocols but has value for identifying persistent deficits.

The use of CAP tests may be particularly useful in settings with limited resources such as LMICs, where monitoring via comprehensive neuropsychological evaluations is often not feasible and barriers to accessing follow-up care are significant. In the acute setting, the implementation of a standard protocol prior to discharge using CAP testing could provide valuable data regarding functional ability, without a significant resource burden. Furthermore, CAP testing could provide information about deficits and deficit severity, which may guide the level of care needed and the types of interventions recommended. Benefits extend beyond care for individuals, as the prevalence of CAP deficits in msTBI could inform and improve resource allocation more broadly within healthcare, particularly in settings where access to both assessment and intervention is limited.

The current study has some limitations. Participants were recruited in collaboration with Muhimbili University of Allied and Health Sciences located in Dar es Salaam Tanzania. While this setting provided information regarding the TBI population in a sub-Saharan African country, which is a significant addition to the literature, it also limits generalizability of results. Further study of the TBI-CAP relationship should be conducted in additional cultures. Further, the sample size was relatively small, and the range of time since injury was very wide. Future efforts should replicate and extend current methods with a larger and more diverse sample, but with a more constricted range of time since injury. Peripheral hearing ability greater than 8 kHz was not tested, which could have affected CAP performance in both the TBI and control groups. While participants with msTBI in this study demonstrated central auditory processing deficits compared to healthy controls, tying those outcomes definitively to the injury remains challenging given the potential impact of other possible contributing factors occurring between injury and evaluation in the context of remote injury. Because systematic management for TBI is often lacking in sub-Saharan Africa, care for patients with TBI may not be optimal (Mwita et al., 2016), and the poor outcomes on CAP tests may be tied to a lack of medical and interventional response following an initial TBI rather than an effect of the TBI itself. Additionally, while the significant group differences and predictive group membership capabilities observed regarding CAP performances certainly support a relationship between history of TBI and CAP test performance, further research is necessary to understand the precise mechanism of this connection.

## Conclusion

The current results expand extant literature correlating CAP test performance with a history of TBI, suggesting the utility of using these measures to assess brain function in individuals with CNS pathology, particularly TBI. Further, results suggest sensitivity of speech-in-noise tests for identifying residual deficits in individuals with a history of TBI. The findings suggest CAP tests may be able to identify persistent dysfunction following TBI, which could be used prospectively, to track cognitive status and long-term outcomes.

## Data Availability

The raw data supporting the conclusions of this article will be made available by the authors, without undue reservation.
